# Local therapy with CpG motifs in a murine model of allergic airway inflammation in IFN-β knock-out mice

**DOI:** 10.1186/1465-9921-6-25

**Published:** 2005-03-05

**Authors:** Victor Matheu, Alexandra Treschow, Ingrid Teige, Vaidrius Navikas, Shohreh Issazadeh-Navikas

**Affiliations:** 1 Section of Medical Inflammation Research, Department of Cell & Molecular Biology; Lund University; Sweden; 2 Fundación Rafael Clavijo de Investigación Biomédica, Tenerife, Spain

**Keywords:** IFN-β, CpG motifs, allergy, asthma, inflammation, synovitis, arthritis, eosinophil, IFN-γ, Th1-response, knockout, lung

## Abstract

**Background:**

CpG oligodeoxynucleotides (CpG-ODN) are capable of inducing high amounts of type I IFNs with many immunomodulatory properties. Furthermore, type-I IFNs have been proposed to play a key role in mediating effects of CpG-ODN. The precise role of IFN-β in the immunomodulatory effects of CpG-ODN is not known.

**Objective:**

Here, we aimed to elucidate the role of IFN-β in the anti-allergic effect of CpG motifs.

**Methods:**

We assessed the immune response in OVA-primed/OVA-challenged IFN-β knockout (-/-) mice compared to wild type (WT) control, after intranasal and systemic treatment with synthetic CpG motifs.

**Results:**

Vaccination with CpG-ODN reduced the number of cells in airways of OVA-sensitized WT but not IFN-β-/- mice. Although airway eosinophilia was reduced in both treated groups, they were significantly higher in IFN-β^-^/- mice. Other inflammatory cells, such as lymphocytes and macrophages were enhanced in airways by CpG treatment in IFN-β-/- mice. The ratio of IFN-γ/IL-4 cytokines in airways was significantly skewed to a Th1 response in WT compared to IFN-β^-^/- group. In contrast, IL-4 and IgE were reduced with no differences between groups. Ag-specific T-cell proliferation, Th1-cytokines such as IFN-γ, IL-2 and also IL-12 were significantly lower in IFN-β-/- mice. Surprisingly, we discovered that intranasal treatment of mice with CpG-ODN results in mild synovitis particularly in IFN-β-/- mice.

**Conclusion:**

Our results indicate that induction of Th1 response by therapy with CpG-ODN is only slightly and partially dependent on IFN-β, while IFN-β is not an absolute requirement for suppression of airway eosinophilia and IgE. Furthermore, our finding of mild synovitis is a warning for possible negative effects of CpG-ODN vaccination.

## Introduction

Allergic diseases are characterized by elevated serum IgE, an inflammatory reaction with increased number of eosinophils, mast cells and an adaptative immune responses orchestrated by Th2-like CD4+ memory T cells secreting an array of cytokines such as IL-4, IL-5 and IL-13. Thus, there are major efforts focused on a therapeutic treatment which will decrease the Th2 profile and/or re-direct the immune response from a Th2, IgE-mediated allergic hypersensitivity reaction towards the more favorable Th1 response. IL-12 and IFN-γ are of primary importance in modulating the Th1/Th2 balance. IFN-γ has been shown to attenuate eosinophil recruitment[1], and also inhibit the development of secondary allergic response [2-4]. There has also been extensive research into therapeutic use of IL-12[5]. However, difficulties with precise dosing and toxicity associated with the direct administration of these cytokines may preclude their therapeutic application.

Another approach is to use natural up-regulators to elevate endogenous levels of IL-12 or IFN-γ. Many microbial products, including heat-killed bacteria and CpG motifs can up-regulate Th1 cytokines. Oligodeoxynucleotides (ODN) containing unmethylated cytosine-guanine motifs (CpG) have powerful immunomodulatory activity in human and murine lymphocytes in both Th1 and Th2 associated diseases [6-12]. It is believed that CpG exert their effect through antigen presenting cells by inducing cytokines such as TNF-alpha, IL-12, IL-18, and IFNs [9,13,14].

Type I IFNs have been proposed as mediators of immunomodulatory effects of CpG oligonucleotides [15]. Importantly, some studies have suggested that endogenous type I IFN might contribute to the downregulation of eosinophil infiltration in murine asthma model [16]. Furthermore, reduced inflammatory infiltration and IgE production have been shown after administration of recombinant IFN-β[17,18]. We have recently demonstrated that lung eosinophilic inflammatory response was exacerbated by the lack of IFN-β gene[19]. Even though it is believed that immunomodulatory effects of CpG-ODN are mediated by type I IFNs, the relative role of IFN-β has not been defined.

In this report, we examined the role of IFN-β in the immune response after CpG treatment in a murine model of allergic inflammation. Our results indicate that induction of Th1 response by therapy with CpG-ODN is partially dependent on IFN-β, while IFN-β is not an absolute requirement for suppression of eosinophilia and IgE.

## Materials and methods

### Animals

Groups of pathogen-free female[20,21] 8-10-week-old, 17-20 g, B10.RIII mice (n = 5 mice per group) were used in the experiments. IFN-β deficient mice (IFN-β-/-) were kindly provided by Dr Leanderson[22]. Genotyping of the offspring has been described before[23]. All animal care and experimentation were conducted at the animal unit of Medical Inflammation Research in Lund in accordance with the current protocols in Lund University.

### Induction of disease and treatment protocol

Immunization and allergen challenge of the mice were carried out according to a short term allergy model protocol by Sur and colleagues [24] with slight modification. Mice were sensitized by i.p. injection on days 0 and 4 with OVA 50 μg (Sigma Chemical Co., St Louis, Mo), with 5 mg alum (Sigma Chemical Co.). At day 14 and 16 after immunization, mice were challenged with 50 μg of OVA plus 5 μg of CpG-ODN (Scandinavian Gene Synthesis AB, Köping, Sweden) delivered through the airways as intranasal drops after light anesthesia. Control mice were immunized with 5 mg alum with PBS, and challenge with PBS using the same schedule as OVA immunized mice. Our previous studies have confirmed that control mice did not show any remarkable allergy changes[19]. The ODNs were designed using published sequences[8,25] consisting of a single-stranded phosphorothioate-modified ODNs with 22 bases containing two CpG motifs (5'-TGACTGTGAACGTTCGAGATGA-3'), highly purified with undetectable levels of LPS (detection limit: 1 ng/mg DNA): and were dissolved in PBS with a final concentration of 1 μg/μl [11]. Mice received either 5 μg of CpG-ODN in PBS or PBS alone intranasally in conjunction with OVA challenges. On day 17 (i.e. 24 h after the last challenge) mice were assessed for lung allergic inflammatory response.

In the prevention study (vaccination), mice were pretreated i.p. with 5 μg of CpG-ODN in PBS on day 0. On the same day, mice were sensitized by i.p. injection with OVA complexed with 5 mg alum (Sigma). On day 4 mice were injected i.p. OVA (50 μg) in Alum (5 mg). On days 14 and 16 after immunization mice were challenged with 50 μg of OVA delivered through the airways as intranasal drops after light anesthesia. On day 17 mice were assessed for lung allergic inflammatory response, 24 hours (h) after the last challenge.

### Bronchoalveolar lavage Fluid (BALF)

Mice were deeply anesthetized with an ip injection of 0.2 ml avertin (20 mg/ml; 2,2,2 tribromoethanol, Sigma-Aldrich) and sacrificed 24 hours after the last OVA exposure. After thoracotomy, the trachea was cannulated and BAL was collected twice with 0.5 mL of PBS and the collected fluid was pooled. Total cell counts were determined using an automated hemocytometer (Sysmex CDA-500, Toa Medical Electronics CO., Ltd., Kobey, Japan), and the fluid was centrifuged (1.000 rpm, 4°C, 10 min). The supernatant was used to determine the airway cytokine and IgE levels contents. The cells were applied to slides using a cytospin apparatus (Auto-smear CF-12DE, Sakura Finetek Europe BV, Zoeterwoude, The Netherlands) and were stained with May-Grunwald-Giemsa staining. Eosinophils were specifically detected by histochemical staining of cyanide-resistant eosinophil peroxidase activity (CREPA) using as substrate 3,3 diaminobenzidine tetrahydrochlorhid (DAB), as described before[26]. Briefly, samples were dried overnight at room temperature and fixed with 4% paraformaldehide for 5 min and PBS for 2 min. Then, samples were incubated in PBS buffer with DAB 60%, H_2_O_2 _30% and NaCN 120% for 7 min. After washing with PBS, samples were counterstained with hemtoxiline 30" and mounted with Kaiser medium (Merck, Darmstadt, Germany). Eosinophils were easily detected by its dark brown color. The slides were examined by light microscopy (×40 magnification) in a blinded fashion counting at least 400 cells per slide

### Allergen specific T cell proliferation

At the time of sacrifice spleens were dissected and a single cell suspensions from each mouse was prepared in DMEM with glutamax I (Gibco BRL, Life Technologies), supplemented with 10% heat-inactivated fetal calf serum, 10 mmol/l HEPES, 50 mmol/l β-mercaptoethanol, 100 U/ml penicillin G, and 100 μg/ml streptomycin. Cells were cultured (5 × 10^6^/ml) in triplicates in 96-well flat-bottomed plates at 37°C, 5% CO2 in a humidified incubator. Cells were cultured in absence or presence of OVA (111 μM), CpG-ODN (1 μg/ml) or concavalin A (4 μg/ml). ^3^H-thymidine (100 μCi/ml) was added 54 h later, and after a further 18 hr later incubation, a beta-scintillation counter measured incorporation.

### Cytokine Assays

Splenocytes were isolated as described and incubated for 48 h with or without OVA (Sigma-Aldrich) (111 μM) in 48-well plates. Enzyme immunoassays were performed as described before[23,27] using monoclonal Ab (anti-IL-2, anti-IL-4, anti-IL-5, anti-IL-12, anti-IFN-γ (BD Pharmingen, San Diego, CA, USA) and reading by chemiluminescence (Victor^®^; 1420 Multilabel Counter ^©^, Wallac Oy; EG & G Turku, Finland).

### Determination of total and OVA-specific IgE levels

Mice were bled at the time of sacrifice. A sandwich ELISA (BD Pharmingen) was used to measure levels of IgG and IgE as described previously[28]. To determine OVA-specific IgE plates were incubated with OVA 10 μg/ml in PBS buffer (pH 7.'5). Procedure was the same as total IgE. Standard curve was performed with sera with known levels of specific IgE as it has been published before [29]. Briefly, real concentration of specific IgE in ng/ml of a pooled serum was determined indirectly by absorption of 50 μl of serum with either conjugated BSA in Sepharose (Pharmacia, Uppsala, Suecia) or conjugated OVA in Sepharose. Total IgE ELISA, as mentioned before, determined the level of not absorbed specific IgE. The percentage of OVA-specific IgE was calculated by reciprocal value of: (IgE not absorbed by OVA-Sepharose/IgE not absorbed by BSA-Sepharose) × 100. The result of a pool of sera from several immunized mice by this method was 402 ng/ml of OVA-specífica IgE. In next experiments this serum was used as standard pattern. For that, plates were coated with OVA (10 μg/ml) overnight 4°C and blocked with 1% BSA in PBS 1 h room temperature. The remainder steps were performed as total IgE ELISA, as described before.

### Flow cytometry

At time of sacrifice spleens were removed and a single cell suspension was made, cells were then lysed with 0.84% NH_3_Cl_2 _and washed in PBS with 1% BSA and 0.01% sodium azide. After blocking Fc receptors, using 24.G2 (from our hybridoma collection), cells were stained with the following antibodies (BD PharMingen); PE conjugated anti-B7.1 (clone 16-10A1), FITC conjugated anti-B7.2 (GL1), cytochrome conjugated anti-B220 (RA3-6B2), APC conjugated anti-Thy1.2 (53-2.1), PE conjugated anti-CD4 (H129.19), cytochrome conjugated anti-CD8 (53-6.7). The cells were then analyzed by flow cytometry FACSort (Becton Dickinson, Franklin Lakes, NJ, USA), using the BD Cell-Quest™ Pro, Version 4.0.1 software (Becton Dickinson). Three individuals per time point and group were analyzed. The program then displays the percentage of events, which express the CD86 molecule and this percentage is the compared between the groups.

### Clinical and Histological analysis of joints for arthritis

Seventeen days post CpG-ODN or control vaccination, paws were visually assessed looking for swelling or deformation with redness in one joint, several joints or severe swelling of the entire paw and/or ankylosis[30]. Then, mice were sacrificed and paws were dissected and were fixed in 4% formaldehyde, decalcified with EDTA (for 2–3 weeks), embedded in paraffin, sectioned at 5μm and stained with hematoxylin and erythrosine. Approximately, 20–30 sections were made from each paw (2 paws per mouse, i.e. front and back paws). The sections were then evaluated blindly for pathological changes in joints (synovitis, erosion or destruction)[31].

### Statistic analysis

The significance of changes was evaluated using Mann-Whitney *U *test. Significance was assumed at *p *values ≤ 0.05.

## Results

### Treatment with different dose of intranasal CpG-ODN showed similar results

The percentage of local eosinophils in airways was increased after immunization and challenge with OVA in BALF of WT and IFN-β-/- compared to non immunized mice. Preliminary data with different dose of CpG administered intranasally with OVA (5 μg, 10 μg or 20 μg) to both strain of mice resulted in similar reduction of percentage of infiltrating eosinophils in BALF (Table 1).

**Table 1:** Eosinophils in airways with different dose of intranasal CpG-ODN

**Treatment**	**Genotype**	**Eosinophils**
PBS	B10.RIII	0.5 %
	IFN-β^-^/-	0.7 %
OVA	B10.RIII	55 %
	IFN-β^-^/-	62 %
OVA+CPG 5 μ	B10.RIII	2.1 %
	IFN-β^-^/-	9.2 %
OVA+CPG 10 μ	B10.RIII	1. 9 %
	IFN-β^-^/-	9.4 %
OVA+CPG 20 μ	B10.RIII	1.9 %
	IFN-β^-^/-	9.0 %

B10.RIII/WT (□) and IFN-β^-^/- (■) mice were sensitized to OVA by intraperitoneal injection and subsequently challenged with OVA either alone or with different dose of CpG-ODN by intranasal drops on days 14 and 16. Eosinophil percentage in bronchoalveolar lavage with different dose of intranasal CpG-ODN were similar in all IFN-β^-^/- treated mice.

### Treatment with CpG-ODN inhibits total number of infiltrating cells in airways in WT but not in IFN-β-/- mice

**Figure 1:**
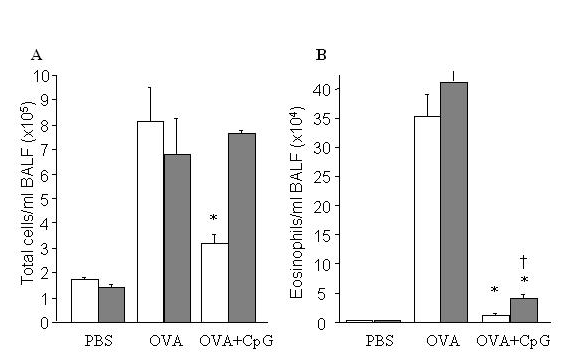
Effects of treatment with CpG-ODN on total BALF cell recruitment (A), eosinophils (B). B10.RIII/WT (□) and IFN-β^-^/- (■) mice were sensitized to OVA by intraperitoneal injection and subsequently challenged with OVA either alone or with CpG-ODN by intranasal drops on days 14 and 16. Cells were harvested on day 17^th^. *n *= 5/group, *P < 0.05 vs. OVA groups. † *P *< 0.05 vs OVA-treated WT mice.

The treatment with 5 μg of CpG administered intranasally with OVA resulted in significant reduction of total number of infiltrating cells in BALF in WT group while it had no effect in IFN-β-/- group (Figure 1A). We examined the number of recruited cells in lung airways after administration of PBS, OVA or CpG-ODN plus OVA and challenge with OVA. We found that OVA nasal challenge increased significantly the number of cells recruited in airways of OVA-primed mice compared to PBS group. CpG-ODN vaccinated mice had reduced the number of cells in OVA-sensitized B10.RIII mice but not in IFN-β-/-.

### Suppression of eosinophilia by CpG-ODN in airways is only partially dependent on IFN-β gene

Next, we were interested in the effect of CpG-ODN treatment on eosinophilia. As expected, we found that OVA-sensitized/OVA-challenge WT mice had a dramatic increase in numbers of eosinophils compared with non-treated WT. Vaccination with CpG-ODN diminished dramatically the number of eosinophils in WT mice while it was only partially effective in prevention of eosinophilia in IFN-β^-^/- mice, and the difference between the CpG-ODN vaccinated and PBS vaccinated mice was statistically significant for both WT and IFN-β^-^/- (figure 1B).

### IFN-γ induction in the airways by CpG-ODNs vaccination is impaired in IFN-β-/- mice

**Figure 2:**
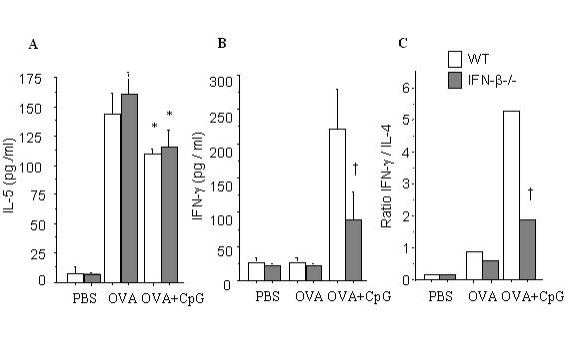
BALF cytokine (protein) concentrations after intranasal CpG-ODN. BALF were collected 24 h after the last challenge from each group (*n *= 5/group) and cytokine levels determined by ELISA in non-immunized, OVA-challenged, and OVA-challenged/CpG-treated B10.RIII (□) and IFN-β^-^/- (■) mice at days 14 and 16. IL-5 (A) levels were significantly augmented after OVA challenge and diminished after CpG vaccination in both strains similarly. IFN-γ (B) was not induced in OVA/primed-OVA/challenge, but was induced after CpG vaccination. IFN-γ was stronger induced in B10.RIII than in IFN-β^-^/- mice. Th1/T2 ratio was stronger skewed to Th1-profile in B10.RIII than in IFN-β^-^/- mice. Data are given as mean ± SEM, *P < 0.05 vs. OVA groups. † *P *< 0.05 vs B10.RIII mice treated with CpG

We were interested in investigating if disease mediated Th2 cytokines or disease counter-acting cytokine, IFN-γ, was effected by the CpG-ODN vaccination. We observed that the level of IL-4 in BALF was reduced from 65 ± 7 pg/ml to 43 ± 6 pg/ml (33% of reduction) in WT mice and from 62 ± 8 pg/ml to 46 ± 87 pg/ml (26%) in IFN-β-/- mice respectively after CpG-ODN vaccination. The levels of IL-5 were significantly reduced in both groups with no difference between groups (figure 2A). IFN-γ production in airways of WT mice was enhanced upon CpG-ODN vaccination and it was dependent on IFN-β gene since its induction was impaired in IFN-β-/- mice (figure 2B). Hence, the ratio IFN-γ/IL-4 determining the Th1/Th2 ratio, was skewed to a Th1 response in both groups although much stronger in WT mice (figure 2C).

### Vaccinated with CpG-ODN induces CD86 expression on B cells in IFN-β-/- mice

**Figure 3:**
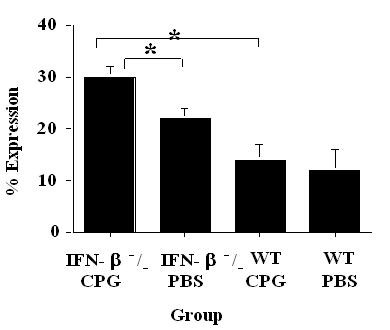
Percent of expression of CD86/B7.2 on B cells in splenocytes of mice at day 17 after immunization and vaccination of ODN-CpG in IFN-β^-^/- mice (KO) and wild type litter-mates (WT).

In order to observe any differences between cell surface markers between IFN-β^-^/- and wild type mice treated with CPG-ODN or with PBS, splenocytes were analyzed by flow cytometry. We could not see any difference in T cell population, in regards to both CD4:CD8 ratio and expression of CD86 (B7.2) on T cells. However, there was a significant difference in CD86 (B7.2) expression on B cells. This difference was observed between CpG-ODN vaccinated IFN-β^-^/- mice and PBS control IFN-β^-^/- mice as well as between CpG-ODN vaccinated IFN-β^-^/- and CpG-ODN vaccinated wild type mice (Figure 3).

### CpG-ODN vaccination induces mild synovitis particularly in IFN-β-/- mice

**Figure 4:**
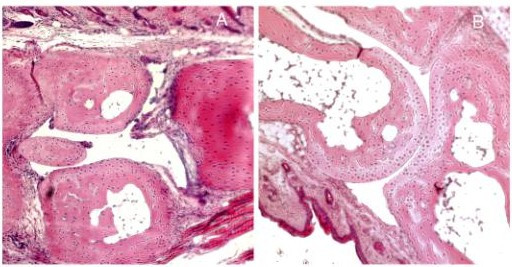
Illustration of joint synovitis after hematoxylin-eosin staining. A. It shows synovitis and pannus formation in IFN-β^-^/- mice treated with CPG-ODN. B. It shows no pathologic changes in a control treated IFN-β^-^/- mice.

Mice did not show any clinical visually deformation. While surveying the capacity of CpG-ODN vaccination to induce IFN-β in different tissues, it was noticeable that there were pathological changes in joints of some mice. Thus, we stained the paws of mice (n = 3) with hematoxylin and erythrosine and evaluated the pathologic changes in joints. Data revealed mild synovitis and pannus formation in multiple joints of CpG-ODN vaccinated mice while no control mice had any pathologic changes. Furthermore, we discovered that mice lacking IFN-β were more affected than their wild type littermates (table 2 and figure 4).

**Table 2:** Histopathologic evaluation of joints for arthritis changes.

**Groups**	**Vaccination**	
	**CpG-ODN**	**Control**

IFN-β^-^/- (n.1)	++	-
IFN-β^-^/- (n.2)	++	-
IFN-β^-^/- (n.3)	+	-
		
WT (n.1)	++	-
WT (n.2)	-	-
WT (n.3)	-	-

Hematoxylin-eosin staining of joints from four different groups of mice (IFN-β^-^/- and their WT littermates with CPG-ODN treatment or control) were analyzed. This revealed mild synovitis and pannus formation in 3/3 IFN-β-/- mice treated with CPG-ODN and 1/3 WT treated with CPG-ODN while no pathological changes were observed in these two non-treated groups.

### Cell profile in airways after vaccination withCpG-ODN

The CpG-ODN vaccination reduced the number of cells in OVA-sensitized B10.RIII mice. However, the number of cells recovered in IFN-β^-^/- mice did not significantly change (table 3). ODN vaccinated mice had a slight increase in numbers of eosinophils compared with non-treated WT. CpG-ODN therapy diminished the number of eosinophils in WT mice, while it was only partially effective in prevention of eosinophilia in IFN-β^-^/- mice with significant differences between the CpG-ODN treated and non-treated mice in WT and IFN-β^-^/- (table 3). Similarly, vaccination with CpG-ODN showed an enhanced response of macrophages in IFN-β^-^/- mice compared to WT mice, but this macrophage response was similar in treated and non-treated WT mice. Lymphocyte and neutrophil response in airways of treated-IFN-β^-^/- mice was also significantly enhanced compared to WT mice.

**Table 3:** Effects of vaccination with CpG-ODN (prevention study) on eosinophil and total BAL cell recruitment.

**Treatment**	**Genotype**	**Total cells**	**Eosinophils**	**Monocytes**	**Lymphocytes**	**Neutrophils**
PBS	B10.RIII	245 ± 43	3 ± 1	232 ± 20	5 ± 1	5 ± 1
	IFN-β^-^/-	259 ± 14	3 ± 1	242 ± 33	6 ± 1	8 ± 2
OVA	B10.RIII	622 ± 37*	381 ± 43*	144 ± 17	62 ± 3*	35 ± 2*
	IFN-β^-^/-	683 ± 66*	427 ± 83*	178 ± 22	55 ± 8*	22 ± 4*
OVA+CpG	B10.RIII	227 ± 18†	2.7 ± 2†	142 ± 19	67 ± 4	14 ± 1
	IFN-β^-^/-	574 ± 32	52 ± 7 † ‡	321 ± 39† ‡	130 ± 38† ‡	70 ± 22† ‡

Cell types quantified in BALF were eosinophils, macrophages, lymphocytes and neutrophils and are expressed as no. of cells × 10^3^/ml. *n *= 5/group, *P < 0.05 vs. untreated groups. † *P *< 0.05 vs OVA-treated mice. ‡ *P *< 0.05 vs WT mice treated with CpG-ODN. OVA-treated mice and control groups.

### Inhibition of OVA-specific IgE in the prevention study (vaccination) by CpG-ODNs is independent of IFN-β

**Figure 5:**
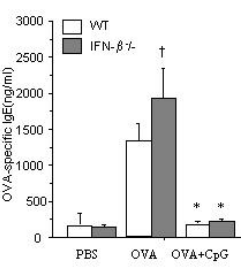
OVA-specific IgE levels in the prevention study (vaccination). B10.RIII/WT (□) and IFN-β^-^/- (■) were sensitized to OVA by intraperitoneal injection either OVA alone or with CpG-ODN and subsequently challenged with OVA by intranasal drops on *days 14 *and *16*; control mice received PBS alone. Cells were harvested on day 17. *n *= 5/group, *P < 0.05 vs. OVA groups. † *P *< 0.05 vs OVA-treated WT mice.

It has been shown that systemic administration of CpG-ODN do not inhibit established IgE response while vaccination inhibits IgE production[32], however the role of INF-β was not investigated. Here, we examined what the function of IFN-β was in prevention of OVA-specific IgE in CpG-ODN vaccine. We found that CpG-ODN vaccine resulted in inhibition of OVA-Specific IgE in both WT and IFN-β-/- mice (figure 5). IgG2a levels were similar in both WT (118 ± 15 μg/ml) and IFN-β-/- (135 ± 25 μg/ml) mice.

### Allergen specific Th1 response as a result of CpG-ODN vaccination is partly impaired in the absence of IFN-β

**Figure 6:**
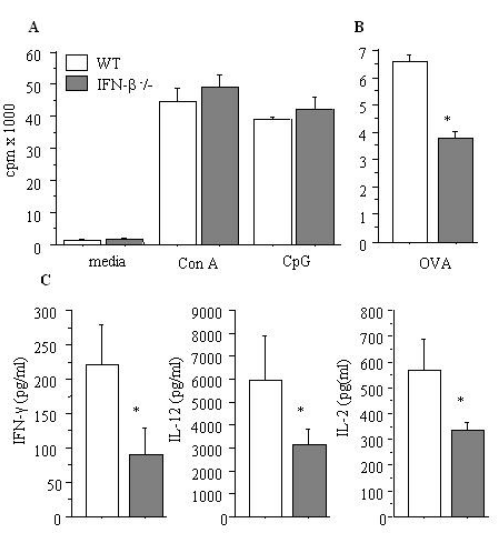
*Ex-vivo *immune response in the prevention study (vaccination). A. *In vitro *stimulation of splenocytes from naïve mice with con A and CpG does not show any difference between B10.RIII (□) and IFN-β-/- mice (■). B. *In vitro *proliferation of OVA restimulated T cells from *in vivo *CpG-vaccinated OVA-primed B10.RIII (□) and IFN-β-/- mice (■). Mice were primed and challenged as in Figure 2. *In vitro *proliferation after recall with OVA was weaker in IFN-β-/- mice (■) than B10.RIII mice (□). C. Th-1 cytokines from supernatants after *in vitro *proliferation of OVA restimulated T cells in OVA-primed/CpG-vaccinated mice. IFN-γ, IL-12 and IL-2 production in supernatants from cell cultures was higher in B10.RIII than in IFN-β-/- mice. *n *= 5/group *P < 0.05 vs. OVA-treated B10.RIII mice.

To address if splenocytes from WT and IFN-β^-^/- respond differently *in vitro*, cells from naïve mice were stimulated and cell proliferation was measured. Splenocytes from both groups, WT and IFN-β^-^/-, had the same proliferation levels after stimulation with concavalin A, CpG or culture media (figure 6A). However, cells from WT immunized mice vaccinated with CpG *in vivo *had more cell proliferation after restimulation with OVA than IFN-β^-^/- immunized and CpG vaccinated mice (figure 6B). Next we assessed whether OVA specific Th1 response, i.e. IFN-γ, IL-2 and IL-12, were affected by CpG-ODN vaccination plus OVA treatment *in vivo*. We found that IFN-γ, IL-12 and IL-2 were significantly lower in OVA-primed/OVA-challenge IFN-β-/- mice compared to WT mice (figure 6C).

## Discussion

Synthetic unmethylated CG dinucleotides within particular sequence context (CpG motifs) mimic bacterial DNA, and are responsible for the immunostimulatory activity of that [6]. CpG oligonucleotides have shown to produce a strong activation of B cells[33], NK cells [34], macrophages[35] and dendritic cells[36] by a direct mechanism. However CpG have also the ability to exert activation of T cells by an indirect mechanism through via IFN-α/β [37,38]. Furthermore, CpG in mice results in production of inflammatory and antiinflammatory cytokines including IL-1, IL-2, IL-6, IL-18, TNF-α, type I IFN (IFN-α/β) and type II IFN (IFN-γ) [39-41]. Type I IFNs (IFN-α/β) have pleiomorphic effect on the immune system with activation of macrophages and stimulation of NK cells to produce IL-12, which in turn induces Th1 cell development[42].

Some of these immunostimulatory effects have been applied in animal models of several diseases including allergic disorders[8,43-50]. It have been shown that therapies using oligonucleotides containing CpG have the ability of immunomodulation with a downregulation of elevated IgE and eosinophilic inflammation in the airways, both of which are orchestrated by cytokines elaborated by Th2 cells. However, systemic administration of CpG has been reported to increase side effects, owing in part to high dose of these oligonucleotides. Systemic immunization, even with adjuvants, induces robust adaptive immune responses at systemic sites but weak in the airways, while local immunization can elicit both systemic and mucosal responses [51-53]. In this report, we have demonstrated that concomitant intranasal administration of low doses of CpG and the offending antigen exerted significant reduction of total number of infiltrating cells, including eosinophils in BALF (table 1).

As mentioned before, CpG in mice results in production of several cytokines including type I IFN (IFN-α/β)[37,38,54-56] which have the ability to exert indirect activation of T cells [37,38]. IFN-β treatment, used by either oral[18] or parenteral[17] via in mice, have shown to produce an inhibition of antigen-induced bronchial inflammation and airway hyperresponsiveness [17,18] probably influenced by the inhibition of Th-2 airway eosinophilia by the suppressive effect on eosinopoiesis [57]. We have recently demonstrated that lung eosinophilic inflammatory response was exacerbated by the lack of IFN-β gene[19]. Even though it is believed that immunomodulatory effects of CpG-ODN may be mediated by type I IFNs [15], the relative role of IFN-β, a type I IFN, has not been defined. Here, we aimed to elucidate whether IFN-β have a key role in the anti-allergic effect of CpG motifs. Our results demonstrate that therapy with CpG-ODN prior to and after the allergen challenge resulted in significant reduction of total number of infiltrating cells, including eosinophils, in BALF in WT mice while CpG-ODN did show an enhanced response of macrophages, lymphocytes and neutrophils in airways of IFN-β-/- mice. These findings might be explained since CpG motifs in bacterial DNA can delay apoptosis of neutrophil granulocytes [58] and macrophages [59], indicating a possibility of inhibition of macrophage apoptosis by CpG and a difference of cellular responses downstream of different Toll-like receptors [59]. Another possibility might be that phosphorothioated ODNs used in our experiments might have been chemoattractants for primary macrophages[60] in the absence of IFN-β. This chemoattractant activity have been exposed as independent of CpG activity[60], since it has not been seen with phosphodiester CpG-ODNs. However, up to our knowledge this is the first reference about the influence of CpG on neutrophils.

It has been shown that systemic administration of CpG-ODN do not inhibit established IgE response while vaccination inhibits IgE production[32]. We found that CpG-ODN vaccine resulted in inhibition of OVA-Specific IgE in both WT and IFN-β^-^/- mice (figure 5). These data underline that IFN-β is not required for the beneficial effect of CpG-ODN vaccine in a model of allergic inflammation. Vaccination with a single low dose of CpG-dinucleotide inhibited OVA-specific IgE production with subsequent upregulation of IgG2a in both groups. The success in inhibiting established IgE response is most likely due to the timing of the protocol where mice received CpG-ODN at the time of priming. This early intervention presumably prevents presence of IgE-plasma cells in the bone marrow as suggested earlier by Peng et al [32].

Production of the Th1 cytokine, IFN-γ, has been reported to be dependent on CpG-ODN-induced IFN-α/β as demonstrated by antibodies that block IFN-α/β[54]. Since, earlier reports target both IFN-α and β, it was unclear if one or both of these cytokines mediate the biological effects of CpG-ODN. In addition, we have recently reported that IFN-β knock out mice do not have any failing mounting a T_H_1 response, measured by IFN-γ production. In contrary, IFN-γ production was significantly elevated as a result of experimental autoimmune encephalomyelitis (EAE), a T_H_1-mediated disease model for multiple sclerosis. Consequently IFN-β knock out mice had more severe and chronic symptoms than their WT littermates with more extensive CNS inflammation and higher demyelination [23]. Thus, here we aimed to investigate the profile of OVA-specific Th1 cytokines after CpG-ODN vaccination in the absence of IFN-β. We found a clear reduction in Th1 response (IL-2 and IFN-γ) in IFN-β knock out mice vaccinated with CpG-ODN which was in agreement with earlier reports[55]. As Th1-promoting activity of CpG-ODN is controlled by IL-12[12], we measured the levels of IL-12 and found that production was elevated in the CpG-ODN WT group. We also found that its induction is partially under the influence of IFN-β triggered by synthetic CpG sequences. Since IFN-γ is almost undetectable in non-treated mice, at least under the conditions used in this study, the results also suggest that CpG is capable of inducing IFN-β in substantial amounts to trigger IFN-γ production. Our findings of Th1 mediated response in systemic immune response were moreover supported by the fact that IFN-γ production was also defective in the inflammatory organ measured in BALF. Moreover, our results also provide evidence that IFN-β is an important cofactor for IFN-γ production through induction of IL-12 pathway as it has been suggested by Sun et al[37] While, it is crucial to underline that IFN-β-/- mice do not have a general defect on mounting a Th1 immune response[23] therefore it is more likely that the defect in inducing a proper Th1 response in IFN-β-/- mice is due to malfunctioning IL-12 and IFN-γ induction through TLR9 pathway as a result of CPG-ODN vaccination. This might also explain the lower proliferative response of OVA-specific Th1 cells in IFN-β-/- mice reported here. Once more, it should be mentioned that IFN-β-/- mice are capable of inducing significantly higher OVA-specific T cell proliferation of Th2 character [19] which might also partly contribute to suppression of a more profound Th1 response. It has been reported that CpG-ODNs do not directly stimulate T cells, but by inducing production of IFN-γ from APCs, thus activating T cells to express CD69 and B7.2[9,37], while their proliferative responses are reduced[37]. It was also shown that CpG stimulate T cells by inducing APCs to synthesize IFN-I, which then act directly on T cells via IFNAR[37]. In addition, it has been suggested that production of type I IFNs by APCs is through increased availability of costimulatory signals on activated DC[37,36]. It has also been reported that stimulation with CpG motifs induces the changes in surface molecules of APCs[25,55,37]. However, the reduced OVA-specific Th1 response in IFN-β-/- mice is less likely to be mediated by lack of upregulation of costimulatory molecules on APCs as we have previously reported that these mice have upregulated B7.1/2 on APCs[19].

After treatment with CPG-ODN we made an interesting observation that the mice developed a mild synovitis, which to our knowledge is the first report of mucosal administration of CPG-ODN causing joint modification. Synovitis is one of the phenotype features of the experimental murine animal models of autoimmune arthritis, such as collagen-induced arthritis (CIA), which is an extensive investigated model of human rheumatoid arthritis. This model can be elicited in susceptible strains by immunization with type II collagen (CII), the major protein of articular cartilage. Assessment of disease includes visual/clinical evaluation of arthritis severity, measurement of humoral and cellular immune responses, including CII-specific antibody titers and T cell responses to CII. In these models, joints are histologically scored for the changes of inflammation including synovitis and periarticular, pannus formation, cartilage damage with marginal erosions or diffuse changes, and bone damage including resorption and periosteal proliferation[31]. It is known that unmethylated CpG-ODN are responsible for induction of arthritis triggered by bacterial DNA[11,61-63] that supports our data. Our finding that mucosal administration of CpG-ODN causes mild synovitis points out a potential hazardous side effect when using CpG-ODN as a treatment.

In summary, we have demonstrated that the CpG-ODNs can partly prevent the development of eosinophilic airway inflammation and allergen specific IgE response in the absence of IFN-β, while Th1 response is defective. In addition, these results demonstrate that mucosal administration of CpG-ODN before allergen exposure could be a less harmful form of active immunotherapy in allergic diseases without impeding systemic immune responses as earlier suggested [8,51]. However, due to the potential of hazardous side effects, meticulous caution must be undertaken prior to considering it as a therapy in allergic asthma.

## Abbreviations

APC: Antigen presenting cells; CpG, cytosine-guanine motifs; ODNs, oligodeoxynucleotides; DAB, 3.3 diamino benzidine tetrahydrochlorhide; BALF, bronchoalveolar lavage fluid; CREPA, (cyanide-resistant eosinophil peroxidase activity); IFNAR, type I IFN receptor; APC, antigen-presenting cells; DC, dendritic cells.

## Authors' contributions

VM conceived of the study, participated in its design and coordination, performed the experiments and drafted the manuscript. AT carried out the analysis of flow cytometry, prepared histological samples of joints and performed the clinical and histological analysis of joints for arthritis. AT and IT generated crossing of IFN-β ko mice to B10.RIII strain of mice, genotyped, backcrossed and maintained the IFN- β-/- mouse line. VN participated in the design and coordination of the study. SI-N participated in the direction of the study, performed histological analysis of joints, as well as writing and preparing the manuscript. All authors read and approved the final manuscript.

## References

[B1] Lack G, Bradley KL, Hamelmann E, Renz H, Loader J, Leung DY, Larsen G, Gelfand EW (1996). Nebulized IFN-gamma inhibits the development of secondary allergic responses in mice. J Immunol.

[B2] Boguniewicz M, Martin RJ, Martin D, Gibson U, Celniker A, Williams M, Leung DY (1995). The effects of nebulized recombinant interferon-gamma in asthmatic airways. J Allergy Clin Immunol.

[B3] Hofstra CL, Van Ark I, Hofman G, Nijkamp FP, Jardieu PM, Van Oosterhout AJ (1998). Differential effects of endogenous and exogenous interferon-gamma on immunoglobulin E, cellular infiltration, and airway responsiveness in a murine model of allergic asthma. Am J Respir Cell Mol Biol.

[B4] Dow SW, Schwarze J, Heath TD, Potter TA, Gelfand EW (1999). Systemic and local interferon gamma gene delivery to the lungs for treatment of allergen-induced airway hyperresponsiveness in mice. Hum Gene Ther.

[B5] Gavett SH, O'Hearn DJ, Li X, Huang SK, Finkelman FD, Wills-Karp M (1995). Interleukin 12 inhibits antigen-induced airway hyperresponsiveness, inflammation, and Th2 cytokine expression in mice. J Exp Med.

[B6] Krieg AM, Yi AK, Matson S, Waldschmidt TJ, Bishop GA, Teasdale R, Koretzky GA, Klinman DM (1995). CpG motifs in bacterial DNA trigger direct B-cell activation. Nature.

[B7] Kline JN, Waldschmidt TJ, Businga TR, Lemish JE, Weinstock JV, Thorne PS, Krieg AM (1998). Modulation of airway inflammation by CpG oligodeoxynucleotides in a murine model of asthma. J Immunol.

[B8] Broide D, Schwarze J, Tighe H, Gifford T, Nguyen MD, Malek S, Van Uden J, Martin-Orozco E, Gelfand EW, Raz E (1998). Immunostimulatory DNA sequences inhibit IL-5, eosinophilic inflammation, and airway hyperresponsiveness in mice. J Immunol.

[B9] Lipford GB, Bauer M, Blank C, Reiter R, Wagner H, Heeg K (1997). CpG-containing synthetic oligonucleotides promote B and cytotoxic T cell responses to protein antigen: a new class of vaccine adjuvants. Eur J Immunol.

[B10] Shirota H, Sano K, Kikuchi T, Tamura G, Shirato K (2000). Regulation of murine airway eosinophilia and Th2 cells by antigen- conjugated CpG oligodeoxynucleotides as a novel antigen-specific immunomodulator. J Immunol.

[B11] Deng GM, Nilsson IM, Verdrengh M, Collins LV, Tarkowski A (1999). Intra-articularly localized bacterial DNA containing CpG motifs induces arthritis. Nat Med.

[B12] Chiaramonte MG, Hesse M, Cheever AW, Wynn TA (2000). CpG oligonucleotides can prophylactically immunize against Th2-mediated schistosome egg-induced pathology by an IL-12-independent mechanism. J Immunol.

[B13] Klinman DM, Yi AK, Beaucage SL, Conover J, Krieg AM (1996). CpG motifs present in bacteria DNA rapidly induce lymphocytes to secrete interleukin 6, interleukin 12, and interferon gamma. Proc Natl Acad Sci U S A.

[B14] Sato Y, Roman M, Tighe H, Lee D, Corr M, Nguyen MD, Silverman GJ, Lotz M, Carson DA, Raz E (1996). Immunostimulatory DNA sequences necessary for effective intradermal gene immunization. Science.

[B15] Hafner M, Zawatzky R, Hirtreiter C, Buurman WA, Echtenacher B, Hehlgans T, Mannel DN (2001). Antimetastatic effect of CpG DNA mediated by type I IFN. Cancer Res.

[B16] Nakajima H, Nakao A, Watanabe Y, Yoshida S, Iwamoto I (1994). IFN-alpha inhibits antigen-induced eosinophil and CD4+ T cell recruitment into tissue. J Immunol.

[B17] Maeda Y, Musoh K, Shichijo M, Tanaka H, Nagai H (1997). Interferon-beta prevents antigen-induced bronchial inflammation and airway hyperreactivity in mice. Pharmacology.

[B18] Satoh Y, Kasama K, Kuwabara M, Diao HY, Nakajima H, Kohanawa M, Minagawa T, Yimin (1999). Suppression of late asthmatic response by low-dose oral administration of interferon-beta in the guinea pig model of asthma. J Interferon Cytokine Res.

[B19] Matheu V, Treschow A, Navikas V, Issazadeh-Navikas S (2003). Upregulation of B7 molecules (CD80 and CD86) and exacerbated eosinophilic pulmonary inflammatory response in mice lacking the IFN-beta gene. J Allergy Clin Immunol.

[B20] Hayashi T, Adachi Y, Hasegawa K, Morimoto M (2003). Less sensitivity for late airway inflammation in males than females in BALB/c mice. Scand J Immunol.

[B21] Yamatomo T, Okano M, Ono T, Nakayama E, Yoshino T, Satoskar AR, Harn DAJ, Nishizaki K (2001). Sex-related differences in the initiation of allergic rhinitis in mice. Allergy.

[B22] Erlandsson L, Blumenthal R, Eloranta ML, Engel H, Alm G, Weiss S, Leanderson T (1998). Interferon-beta is required for interferon-alpha production in mouse fibroblasts. Curr Biol.

[B23] Teige I, Treschow A, Teige A, Mattsson R, Navikas V, Leanderson T, Holmdahl R, Issazadeh-Navikas S (2003). IFN-beta gene deletion leads to augmented and chronic demyelinating experimental autoimmune encephalomyelitis. J Immunol.

[B24] Sur S, Wild JS, Choudhury BK, Sur N, Alam R, Klinman DM (1999). Long term prevention of allergic lung inflammation in a mouse model of asthma by CpG oligodeoxynucleotides. J Immunol.

[B25] Martin-Orozco E, Kobayashi H, Van Uden J, Nguyen MD, Kornbluth RS, Raz E (1999). Enhancement of antigen-presenting cell surface molecules involved in cognate interactions by immunostimulatory DNA sequences. Int Immunol.

[B26] Ten RM, Pease LR, McKean DJ, Bell MP, Gleich GJ (1989). Molecular cloning of the human eosinophil peroxidase. Evidence for the existence of a peroxidase multigene family. J Exp Med.

[B27] Teige A, Teige I, Lavasani S, Bockermann R, Mondoc E, Holmdahl R, Issazadeh-Navikas S (2004). CD1-dependent regulation of chronic central nervous system inflammation in experimental autoimmune encephalomyelitis. J Immunol.

[B28] Matheu V, Navikas V, Issazadeh S (2001). Susceptibility of B10.RIII mouse strain to develop inflammatory allergic pulmonary disease.. Alergol Inmunol Clin.

[B29] Zuberi RI, Apgar JR, Chen SS, Liu FT (2000). Role for IgE in airway secretions: IgE immune complexes are more potent inducers than antigen alone of airway inflammation in a murine model. J Immunol.

[B30] Svensson L, Jirholt J, Holmdahl R, Jansson L (1998). B cell-deficient mice do not develop type II collagen-induced arthritis (CIA). Clin Exp Immunol.

[B31] Johansson AC, Nakken B, Sundler M, Lindqvist AK, Johannesson M, Alarcon-Riquelme M, Bolstad AI, Humphreys-Beher MG, Jonsson R, Skarstein K, Holmdahl R (2002). The genetic control of sialadenitis versus arthritis in a NOD.QxB10.Q F2 cross. Eur J Immunol.

[B32] Peng Z, Wang H, Mao X, HayGlass KT, Simons FE (2001). CpG oligodeoxynucleotide vaccination suppresses IgE induction but may fail to down-regulate ongoing IgE responses in mice. Int Immunol.

[B33] Liang H, Nishioka Y, Reich CF, Pisetsky DS, Lipsky PE (1996). Activation of human B cells by phosphorothioate oligodeoxynucleotides. J Clin Invest.

[B34] Ballas ZK, Rasmussen WL, Krieg AM (1996). Induction of NK activity in murine and human cells by CpG motifs in oligodeoxynucleotides and bacterial DNA. J Immunol.

[B35] Takeshita S, Takeshita F, Haddad DE, Ishii KJ, Klinman DM (2000). CpG oligodeoxynucleotides induce murine macrophages to up-regulate chemokine mRNA expression. Cell Immunol.

[B36] Jakob T, Walker PS, Krieg AM, Udey MC, Vogel JC (1998). Activation of cutaneous dendritic cells by CpG-containing oligodeoxynucleotides: a role for dendritic cells in the augmentation of Th1 responses by immunostimulatory DNA. J Immunol.

[B37] Sun S, Zhang X, Tough DF, Sprent J (1998). Type I interferon-mediated stimulation of T cells by CpG DNA. J Exp Med.

[B38] Rothenfusser S, Hornung V, Krug A, Towarowski A, Krieg AM, Endres S, Hartmann G (2001). Distinct CpG oligonucleotide sequences activate human gamma delta T cells via interferon-alpha/-beta. Eur J Immunol.

[B39] Sun S, Zhang X, Tough D, Sprent J (2000). Multiple effects of immunostimulatory DNA on T cells and the role of type I interferons. Springer Semin Immunopathol.

[B40] Sun S, Sprent J (2000). Role of type I interferons in T cell activation induced by CpG DNA. Curr Top Microbiol Immunol.

[B41] Sprent J, Zhang X, Sun S, Tough D (2000). T-cell proliferation in vivo and the role of cytokines. Philos Trans R Soc Lond B Biol Sci.

[B42] Roman M, Martin-Orozco E, Goodman JS, Nguyen MD, Sato Y, Ronaghy A, Kornbluth RS, Richman DD, Carson DA, Raz E (1997). Immunostimulatory DNA sequences function as T helper-1-promoting adjuvants. Nat Med.

[B43] Spiegelberg HL, Broide D, Tighe H, Roman M, Raz E (1999). Inhibition of allergic inflammation in the lung by plasmid DNA allergen immunization. Pediatr Pulmonol Suppl.

[B44] Spiegelberg HL, Orozco EM, Roman M, Raz E (1997). DNA immunization: a novel approach to allergen-specific immunotherapy. Allergy.

[B45] Spiegelberg HL, Tighe H, Roman M, Broide D, Raz E (1998). Inhibition of IgE formation and allergic inflammation by allergen gene immunization and by CpG motif immunostimulatory oligodeoxynucleotides. Allergy.

[B46] Broide D, Raz E (1999). DNA-Based immunization for asthma. Int Arch Allergy Immunol.

[B47] Broide DH, Paine MM, Firestein GS (1992). Eosinophils express interleukin 5 and granulocyte macrophage-colony- stimulating factor mRNA at sites of allergic inflammation in asthmatics. J Clin Invest.

[B48] Broide DH, Stachnick G, Castaneda D, Nayar J, Miller M, Cho J, Rodriquez M, Roman M, Raz E (2001). Immunostimulatory DNA mediates inhibition of eosinophilic inflammation and airway hyperreactivity independent of natural killer cells in vivo. J Allergy Clin Immunol.

[B49] Broide DH, Stachnick G, Castaneda D, Nayar J, Miller M, Cho JY, Roman M, Zubeldia J, Hayashi T, Raz E (2001). Systemic administration of immunostimulatory DNA sequences mediates reversible inhibition of Th2 responses in a mouse model of asthma. J Clin Immunol.

[B50] Cho JY, Miller M, Baek KJ, Castaneda D, Nayar J, Roman M, Raz E, Broide DH (2001). Immunostimulatory DNA sequences inhibit respiratory syncytial viral load, airway inflammation, and mucus secretion. J Allergy Clin Immunol.

[B51] Shirota H, Sano K, Kikuchi T, Tamura G, Shirato K (2000). Regulation of T-helper type 2 cell and airway eosinophilia by transmucosal coadministration of antigen and oligodeoxynucleotides containing CpG motifs. Am J Respir Cell Mol Biol.

[B52] Magone MT, Chan CC, Beck L, Whitcup SM, Raz E (2000). Systemic or mucosal administration of immunostimulatory DNA inhibits early and late phases of murine allergic conjunctivitis. Eur J Immunol.

[B53] Takabayashi K, Libet L, Chisholm D, Zubeldia J, Horner AA (2003). Intranasal immunotherapy is more effective than intradermal immunotherapy for the induction of airway allergen tolerance in Th2-sensitized mice. J Immunol.

[B54] Krug A, Rothenfusser S, Hornung V, Jahrsdorfer B, Blackwell S, Ballas ZK, Endres S, Krieg AM, Hartmann G (2001). Identification of CpG oligonucleotide sequences with high induction of IFN-alpha/beta in plasmacytoid dendritic cells. Eur J Immunol.

[B55] Cho HJ, Hayashi T, Datta SK, Takabayashi K, Van Uden JH, Horner A, Corr M, Raz E (2002). IFN-alphabeta Promote Priming of Antigen-Specific CD8(+) and CD4(+) T Lymphocytes by Immunostimulatory DNA-Based Vaccines. J Immunol.

[B56] Van Uden JH, Tran CH, Carson DA, Raz E (2001). Type I interferon is required to mount an adaptive response to immunostimulatory DNA. Eur J Immunol.

[B57] Klimpel GR, Fleischmann WRJ, Klimpel KD (1982). Gamma interferon (IFN gamma) and IFN alpha/beta suppress murine myeloid colony formation (CFU-C)N: magnitude of suppression is dependent upon level of colony-stimulating factor (CSF). J Immunol.

[B58] Jozsef L, Khreiss T, Filep JG (2004). CpG motifs in bacterial DNA delay apoptosis of neutrophil granulocytes. Faseb J.

[B59] Kim SO, Ono K, Han J (2001). Apoptosis by pan-caspase inhibitors in lipopolysaccharide-activated macrophages. Am J Physiol Lung Cell Mol Physiol.

[B60] Baek KH, Ha SJ, Sung YC (2001). A novel function of phosphorothioate oligodeoxynucleotides as chemoattractants for primary macrophages. J Immunol.

[B61] Deng GM, Tarkowski A (2001). Synovial cytokine mRNA expression during arthritis triggered by CpG motifs of bacterial DNA. Arthritis Res.

[B62] Miyata M, Kobayashi H, Sasajima T, Sato Y, Kasukawa R (2000). Unmethylated oligo-DNA containing CpG motifs aggravates collagen-induced arthritis in mice. Arthritis Rheum.

[B63] Svelander L, Erlandsson Harris H, Lorentzen JC, Trollmo C, Klareskog L, Bucht A (2004). Oligodeoxynucleotides containing CpG motifs can induce T cell-dependent arthritis in rats. Arthritis Rheum.

